# Genome-Wide Association Study of Pericarp Color in Rice Using Different Germplasm and Phenotyping Methods Reveals Different Genetic Architectures

**DOI:** 10.3389/fpls.2022.841191

**Published:** 2022-03-09

**Authors:** Wu Yang, Luo Chen, Junliang Zhao, Jian Wang, Wenhui Li, Tifeng Yang, Jingfang Dong, Yamei Ma, Lian Zhou, Jiansong Chen, Wei Wu, Shaohong Zhang, Bin Liu

**Affiliations:** ^1^Rice Research Institute, Guangdong Academy of Agricultural Sciences, Guangzhou, China; ^2^Guangdong Key Laboratory of New Technology in Rice Breeding, Guangzhou, China; ^3^Guangdong Rice Engineering Laboratory, Guangzhou, China

**Keywords:** rice, pericarp color, quantitative trait locus, candidate gene, genome-wide association study

## Abstract

Pericarp colors (PC) in rice are determined by the types and content of flavonoids in the pericarp. The flavonoid compounds have strong antioxidant activities and are beneficial to human health. However, the genetic basis of PC in rice is still not well-understood. In this study, a genome-wide association study (GWAS) of PC was performed in a diverse rice collection consisting of 442 accessions using different phenotyping methods in two locations over 2 years. In the whole population consisting of white and colored pericarp rice, a total of 11 quantitative trait loci (QTLs) were identified using two phenotyping methods. Among these QTLs, nine were identified using the phenotypes represented by the presence and absence of pigmentation in pericarp, while 10 were identified using phenotypes of the degree of PC (DPC), in which eight are common QTLs identified using the two phenotyping methods. Using colored rice accessions and phenotypes based on DPC, four QTLs were identified, and they were totally different from the QTLs identified using the whole population, suggesting the masking effects of major genes on minor genes. Compared with the previous studies, 10 out of the 15 QTLs are first reported in this study. Based on the differential expression analysis of the predicted genes within the QTL region by both RNA-seq and real-time PCR (RT-PCR) and the gene functions in previous studies, *LOC_Os01g49830*, encoding a RAV transcription factor was considered as the candidate gene underlying *qPC-1*, a novel QTL with a large effect in this study. Our results provide a new insight into the genetic basis of PC in rice and contribute to developing the value-added rice with optimized flavonoid content through molecular breeding.

## Introduction

Rice is one of the most important crops, serving as the staple food for more than half of the world’s population. For a long time, the majority of rice planted and consumed worldwide is white rice. The grains of most rice cultivars have white pericarps, but some have colored pericarps, such as brown, red, or black (purple). The colored pericarps accumulate abundant flavonoid compounds, which have strong antioxidant activities and are beneficial to human health (Sangma and Parameshwari, 2021). Therefore, colored rice is arousing more interest and attention.

Previous studies suggest that the types and content of flavonoids determine the pericarp color (PC) in rice grains (Joseph et al., 1998; Furukawa et al., 2006). Red and black rice are the most common types of colored rice, which accumulate proanthocyanidins and anthocyanins in their pericarps, respectively (Reddy et al., 1996; Oki et al., 2002). Proanthocyanidins and anthocyanidins are produced through a special branch of the flavonoid pathway, and they share most of the biosynthetic genes, which have been well-studied (Winkel-Shirley, 2001). Some structural and regulatory genes related to their biosynthesis have been isolated, and their functions have been confirmed. So far, several structural genes have been identified in rice, such as the genes encoding chalcone synthase (*OsCHS1* and *OsCHS2*) (Reddy et al., 1996; Shih et al., 2008), chalcone isomerase (*OsCHI*) (Druka et al., 2002), flavanone 3-hydroxylase (*OsF3H*) (Kim et al., 2008), flavonoid 3-hydroxylase (*OsF3′H*) (Shih et al., 2008), dihydroflavonol 4-reductase (*OsDFR*/*Rd*) (Furukawa et al., 2006), anthocyanidin synthase (*OsANS*) (Shih et al., 2008), and UDP-dependent glucosyltransferase (*UGT*) (Ko et al., 2008). These structural genes are predominantly regulated by the MYB (v-myb avian myeloblastosis viral oncogene homolog), bHLH (basic helix-loop-helix) and WD40 (tryptophan-aspartate repeats) transcription factors (Koes et al., 2005; Morishita et al., 2009; Hichri et al., 2011; Kim et al., 2011; Czemmel et al., 2012; James et al., 2017; Lloyd et al., 2017).

Classical genetic analysis indicated that two genes, *Pb* on chromosome 4 and *Pp* on chromosome 1, are required for the pericarp pigmentation with anthocyanins in black rice (Causse et al., 1994; Yoshimura et al., 1997), while red pericarp is controlled by two major genes, *Rd* on chromosome 1 and *Rc* on chromosome 7 (Furukawa et al., 2006; Sweeney et al., 2006). *Rd* encoding a dihydroflavonol-4-reductase and *Rc* encoding a bHLH domain-containing transcription factor are involved in the synthesis of proanthocyanidins. The loss-of-function allele, *rc*, characterized by the 14-bp deletion in *Rc* that causes a frameshift mutation and a premature stop codon, results in the pericarp pigmentation from red to white. The *Rcrd* genotype produces brown pericarp, while *RcRd* produces red pericarp. Introduction of *Rd* into *Rcrd* rice changes the PC from brown to red, while *rcRd* or *rcrd* produces the white pericarp (Furukawa et al., 2006; Sweeney et al., 2006). So far, studies have identified several allelic variations in *Rc* that cause the changes in pericarp pigmentation in rice. Except for the 14-bp deletion resulting in a frameshift mutation, one-base transversion also causes premature termination of the Rc protein, leading to white rice (Sweeney et al., 2006; Gross et al., 2010), while a G base deletion in conjunction with the 14-bp deletion restores the reading frame of the gene and reverted the pericarp pigmentation to red (Brooks et al., 2008; Lee et al., 2009).

The development of molecular marker technology provides a powerful tool for dissecting the genetic basis of quantitatively inherited traits. A few QTLs associated with PC in rice have been reported using biparental QTL analysis (Sweeney et al., 2006; Wang and Shu, 2007; Dong et al., 2008; Maeda et al., 2014). With the rapid development of genome sequencing technology, a genome-wide association study (GWAS) based on linkage disequilibrium (LD) in a diverse natural population and using highly dense markers, such as single-nucleotide polymorphisms (SNPs), has been developed and proved to be a powerful approach for identifying genes that control complex traits, such as PC in rice, on a large scale (Wang et al., 2020). Using the GWAS approach, more QTLs for PC were identified in rice (Huang et al., 2010; Shao et al., 2011; Wang et al., 2016; Yang et al., 2018; Wang et al., 2020). Huang et al. (2010, 2011) identified three QTLs associated with PC and two genes, *Os02g0650900* and *Os08g0301500*, encoding glutamate dehydrogenase and sucrose-phosphate synthase, were considered to be candidate genes underlying QTLs on chromosomes 2 and 8, respectively. Wang et al. (2016) identified a new QTL for PC, *qPc10*, which is unique to Aus, and the gene *Os10g0536400*, encoding flavanone 3-hydroxylase (F3H), was the candidate gene underlying *qPc10*. F3H catalyzes the reaction from flavanone to dihydroflavonol, the first committed step of the biosynthesis of proanthocyanidins and anthocyanins (Kim et al., 2008). In another study, 10 QTLs for PC were detected, and MYB, bHLH, and WD transcription factors were speculated as candidate genes for the corresponding QTLs (Yang et al., 2018). Although significant progress has been made in the discovery of QTLs or genes associated with PC in rice, only a few functional genes have been identified. In addition, the masking effect of major genes on QTLs with minor effects is common in complex traits in rice, such as disease resistance (Yu et al., 2003; Liu et al., 2004) and yield-related traits (Zhong et al., 2011). However, rare studies have considered this problem. The QTLs that truly contribute to the degree of PC (DPC) might not be identified because of the masking effect of major genes.

To address the above issues, 442 rice accessions from 61 countries, which were selected from the Rice Diversity Panel 2 (RDP2) and genotyped by 700K SNPs (McCouch et al., 2016), were used for genetic dissection of PC in two locations over 2 years in this study. To identify the QTLs that determine the presence and absence of PC, and the QTLs for the degree of PC (DPC), two different phenotyping methods were used, i.e., presence and absence of pigmentation in the pericarp (PA method) and DPC by comparing with a visual card (DPC method). To eliminate the masking effect of the major genes and identify the QTLs controlling DPC, the colored rice accessions and their DPC were further used for GWAS. Our results suggest that the environment does not affect the presence and absence of PC in rice but the DPC. In total, fifteen QTLs for PC were identified through GWAS in the whole population and the colored rice accessions. Among them, 10 QTLs were identified for the first time in this study, and the other five QTLs were co-localized with the previously identified QTLs or genes including *Rc*. Interestingly, four QTLs for DPC identified in the colored rice accessions were totally different from those identified in the whole population using phenotype of either DPC or presence and absence of pigmentation, suggesting that there exist masking effect of major genes on QTLs controlling DPC in rice. Based on gene differential expression analysis, gene annotation, and literature, *LOC_Os01g49830*, encoding a RAV transcription factor, was considered as the candidate gene underlying *qPC-1*, a novel QTL with a large effect in this study. This study provides new insight into the genetic basis of PC in rice and contributes to molecular breeding for colored rice.

## Materials and Methods

### Plant Materials

A subset of the RDP2 consisting of 442 rice accessions from 61 countries was used for GWAS in this study (Supplementary Table 1). These rice accessions were selected from the RDP2 consisting of 1,568 rice accessions based on their origins and diversity, including three groups of *Indica* (218 accessions), *Japonica* (148 accessions), and *Aus* (76 accessions) (McCouch et al., 2016).

### Evaluation of Pericarp Colors

**FIGURE 1:**
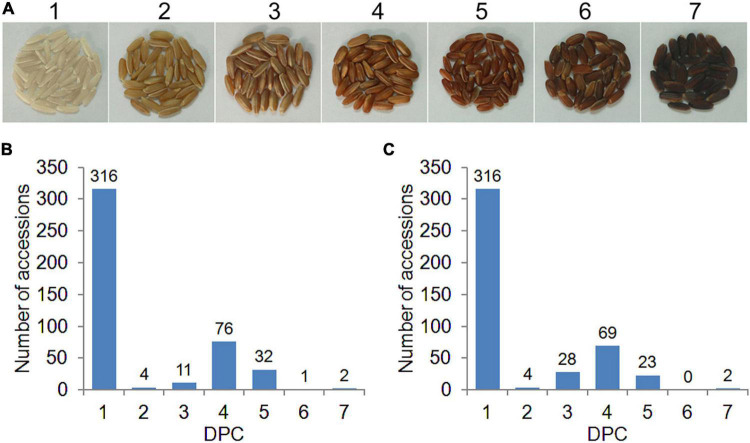
Distribution of pericarp color (PC) in 442 rice accessions. **(A)** The visual card with scores from 1 (white) to 7 (dark brown) is based on the degree of PC (DPC) from light to dark. **(B,C)** Distribution of DPC in 2016GZ and 2018YJ, respectively.

The 442 rice accessions were planted in the experimental bases of Guangzhou (2016GZ) and Yangjiang (2018YJ) in Guangdong Province, China, in the second cropping season in 2016 and 2018, respectively. The experiments were arranged in a randomized complete block design with two replicates. The field management, including irrigation, fertilization, and disease and pest control, followed the conventional practice in rice production. At complete maturity, rice grains for each accession were harvested and dried naturally. A total of 50 healthy grains for each accession were randomly selected and carefully hulled. In this study, two phenotyping methods were used to evaluate the PC. First, the PC of each accession was evaluated based on the presence (1) or absence (0) of pigmentation in the pericarp (PA method). To better exhibit the PC variations in the diverse rice panel, we developed a visual card with scores from 1 (white) to 7 (dark brown), according to the DPC from light to dark (DPC method, Figure 1A). Then the DPC was assessed by comparing with the visual card and given a score for each accession (Figure 1A). DPC of each accession was scored separately by three individuals, and the consistent scores were used for GWAS analysis.

### Genome-Wide Association Study Analysis and Quantitative Trait Loci Delimitation

Genome-wide association study analysis was performed as described in our previous study (Zhao et al., 2018) by using GAPIT version 2 software (Tang et al., 2016) and high-density rice array (HDRA) dataset consisting of 700K SNPs (McCouch et al., 2016). Briefly, SNPs were filtered using the criteria of having less than 30% of missing data and minor allele frequency (MAF) > 0.05 (McCouch et al., 2016). GWAS was conducted using the mixed linear model (MLM) with kinship matrix, and the principal component was set to 3 in GAPIT. R-package qqman (Turner, 2014) was used to produce Manhattan and QQ plots. A region having two or more than two significant SNPs (*p* < 0.0001) (Zhao et al., 2018; Yang et al., 2021) within 160 kb is considered as one QTL, according to 80 kb of the LD decay in the population used in this study (Supplementary Figure 1).

### RNA-Sequencing

Four lines with consistent flowering time, including two lines with white pericarp (accessions 611 and 689) and two lines with red pericarp (accessions 414 and 752), were selected based on haplotype analysis to conduct RNA-sequencing. The spikes were sampled on the15th day after flowering, and total RNA was extracted from spikes using Trizol reagent (Invitrogen). RNA-seq was performed using Annoroad Gene Technology (Beijing, China), and data analysis was conducted using the HISAT2-Stringtie-Deseq2 pipeline (Pertea et al., 2016). Raw counts of each sample exported from Stringtie were imported and normalized by DEseq2. Genes with read count less than 30 were regarded as having no expression. Then, differentially expressed genes between two sets of contrasting samples were identified according to the criteria of *p*-value ≤ 0.05 and fold change ≥2 or ≤0.5 between white and red pericarp.

### Real-Time PCR Analysis

cDNA synthesis was performed using the PrimeScript™ RT reagent kit (Takara, Japan). The PCR analysis was performed using the BioRad CFX 96 system. The primers were designed using the Primer designing tool on NCBI.^1^ The EF1α was used as the normalized genes for mRNA. The same RNA samples used in RNA-Seq assays were used to confirm the results of RNA-Seq, and all reactions were repeated three times. The gene-specific primers used in this study are listed in Supplementary Table 3.

## Results

### Phenotypic Variations of Pericarp Colors in 442 Rice Accessions

To better exhibit the PC variations of the diverse rice panel and identify the QTLs associated with DPC, we developed a visual card with scores of 1–7 based on the DPC from light to dark (Figure 1A). According to the scores of DPC, the whole population used in this study consisted of 316 white accessions (score 1) and 126 colored accessions (score 2–7), and the DPC in the 442 accessions displayed a continuous distribution. Large variations in DPC were observed in the colored rice accessions, ranging from score 2 to 7 (Figures 1B,C). Interestingly, the DPC of white rice accessions remained the same (score 1), while the DPC of about half of the colored rice accessions varied across environments (2016GZ and 2018YJ) (Supplementary Table 1 and Figures 1B,C). A highly and a moderately positive correlation was observed between the DPC in 2016GZ and 2018YJ in the whole population (*r* = 0.9627, *p* < 0.0001) and colored population (*r* = 0.5825, *p* < 0.0001), respectively, indicating that the grain pericarps with or without pigment in rice are highly hereditary, while the quantitative inheritance of DPC is affected by the environment.

### Identification of Quantitative Trait Loci for Pericarp Colors by Genome-Wide Association Study

Based on the criteria of having less than 30% of missing data and MAF more than 5% in the whole population, 572,690 SNPs were selected for GWAS from the 700K SNP dataset in the Open Rice GWAS Platform (McCouch et al., 2016). GWAS of PC was conducted using the whole and the colored population, and phenotypes were generated using different phenotyping methods, respectively. According to the LD decay analysis, the LD distance of the population used in this study is about 80 kb (Supplementary Figure 1). Thus, a QTL was declared when there are two or more significant SNPs (Zhao et al., 2018; Yang et al., 2021) (*p* < 0.0001) within a 160-kb region centered on the most significant SNP.

**FIGURE 2:**
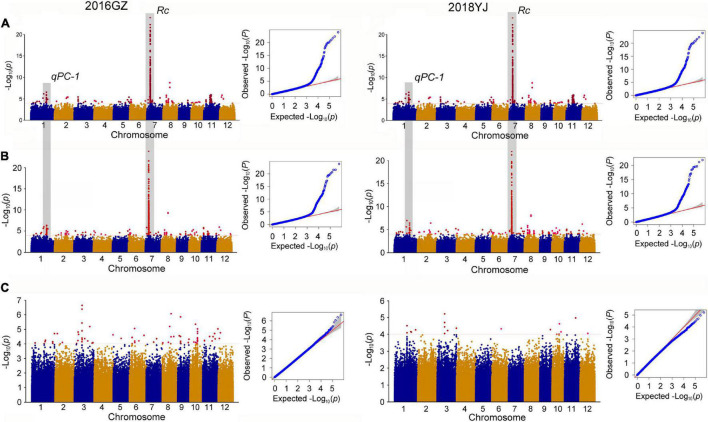
Genome-wide association study for PC. **(A,B)** Manhattan plots of GWAS in 12 chromosomes and QQ plots of *p*-values conducted in the whole population using the PA and DPC phenotyping method, respectively. PA method, pericarp color were evaluated based on presence or absence of pigmentation; DPC method, pericarp color were evaluated based on the degree of pericarp color (DPC) using the visual card with scores of 1–7. **(C)** Manhattan plots of GWAS in 12 chromosomes and QQ plot of *p*-value conducted in the colored population.

In the whole population consisting of white and colored rice, a total of 11 QTLs were identified in two environments (2016GZ and 2018YJ) using two phenotyping methods: the presence and absence of pigmentation in the pericarp (PA method) and DPC method. Among these QTLs, nine QTLs (*qPC-1*, *qPC-3*, *qPC-6*, *qPC-7*, *qPC-8a*, *qPC-8b*, *qPC-8c*, *qPC-9*, and *qPC-11*) located on chromosomes 1, 3, 6, 7, 8, 9, and 11 were identified using the PA method, and all of them were identical in two locations over 2 years (2016GZ and 2018YJ) (Figure 2A and Table 1), while 10 QTLs were identified using the DPC method, in which *qPC-2* and *qPC-8d* were only detected using the DPC method in 2016GZ and 2018YJ, respectively, and other eight QTLs (*qPC-1*, *qPC-6*, *qPC-7*, *qPC-8a*, *qPC-8b*, *qPC-8c*, *qPC-9*, and *qPC-11*) were detected in both environments (2016GZ and 2018YJ) (Figure 2B and Table 1). Notably, these eight QTLs were also identified using the PA method, and *qPC-7*, which overlapped with the major gene *Rc*, had the largest contribution to the phenotypic variations in the whole population.

**TABLE 1:** QTLs for pericarp color identified in this study.

QTL	Environment^†^	Evaluation method*	Chromosome	The most significant SNP position (bp)^#^	*p*-value	Variation explained (%)	Co-location QTL/Candidate genes^§^	References
**Whole population**								
*qPC-1*	2016GZ	PA	1	28,560,888	2.68E-07	3.61		
		DPC		28,560,888	4.43E-07	3.69		
	2018YJ	PA	1	28,560,888	2.68E-07	3.61		
		DPC		28,560,888	3.87E-07	4.25		
*qPC-2*	2016GZ	DPC	2	20,137,220	3.41E-05	2.46		
*qPC-3*	2016GZ	PA	3	7,256,887	3.49E-06	2.92		
	2018YJ	PA	3	7,256,887	3.49E-06	2.92		
*qPC-6*	2016GZ	PA	6	2,829,129	7.79E-05	2.10		
		DPC		2,829,129	2.63E-05	2.53		
	2018YJ	PA	6	2,829,129	7.79E-05	2.10		
		DPC		2,829,129	5.15E-05	2.67		
*qPC-7*	2016GZ	PA	7	6,105,212	1.19E-24	15.72	*Rc*	Sweeney et al., 2006
		DPC		6,105,212	1.25E-24	16.66		
	2018YJ	PA	7	6,105,212	1.19E-24	15.72		
		DPC		6,218,264	1.17E-22	17.19		
*qPC-8a*	2016GZ	PA	8	6,095,179	1.47E-06	3.15		
		DPC		6,095,179	5.63E-06	2.96		
	2018YJ	PA	8	6,095,179	1.47E-06	3.15		
		DPC		6,095,179	1.51E-06	3.8		
*qPC-8b*	2016GZ	PA	8	9,415,989	2.05E-05	2.45		
		DPC		9,526,822	2.08E-05	2.6		
	2018YJ	PA	8	9,415,989	2.05E-05	2.45		
		DPC		9,526,822	1.38E-05	3.09		
*qPC-8c*	2016GZ	PA	8	12,397,795	1.70E-09	5.01	*Os08g0301500*	Huang et al., 2010
		DPC		12,396,756	4.47E-10	5.71		
	2018YJ	PA	8	12,397,795	1.70E-09	5.01		
		DPC		12,397,795	7.29E-09	5.57		
*qPC-8d*	2018YJ	DPC	8	17,842,687	1.87E-05	2.99	*RM339* ^⋇^	Shao et al., 2011
*qPC-9*	2016GZ	PA	9	16,851,795	2.30E-05	2.42		
		DPC		16,851,795	1.44E-05	2.70		
	2018YJ	PA	9	16,851,795	2.30E-05	2.42		
		DPC		16,851,795	3.81E-05	2.77		
*qPC-11*	2016GZ	PA	11	4,082,549	1.84E-05	2.48		
		DPC		4,082,549	1.64E-06	3.31		
	2018YJ	PA	11	4,082,549	1.84E-05	2.48		
		DPC		4,236,188	9.38E-06	3.21		
**Colored population**								
*qPC_C-3*	2016GZ	DPC	3	14,087,316	2.30E-07	25.24		
	2018YJ	DPC	3	14,032,259	6.13E-06	16.53		
*qPC_C-6*	2016GZ	DPC	6	5,129,068	3.63E-05	15.43	*OsC1*	Gao et al., 2011; Hu et al., 2020
*qPC_C-9*	2016GZ	DPC	9	5,331,163	1.42E-06	21.6	*qDRC-9*	Dong et al., 2008
*qPC_C-10*	2016GZ	DPC	10	13,761,244	9.45E-06	17.94		

*^†^The experimental bases of Guangzhou in 2016 (2016GZ) and Yangjiang in 2018 (2018YJ).*

*Evaluation of pericarp color based on the presence and absence of pigmentation in the pericarp (PA) and degree of pericarp color (DPC).

*^#^Position of the most significant SNP at the QTL region.*

*^§^ QTLs or candidate genes for PC identified by the previous studies.*

*^⋇^Simple sequence repeat marker associated with grain color and flavonoid content.*

To eliminate the masking effects of major genes on minor genes or minor effect QTLs and identify the QTLs controlling DPC, we removed the white rice accessions and used the colored rice and their DPC for GWAS. Indeed, the major gene *Rc* responsible for the production of pigment in rice (Furukawa et al., 2006; Sweeney et al., 2006) was not detected, but four QTLs for DPC were detected in this colored population (Figure 2C and Table 1). The four QTLs were mapped on chromosomes 3, 6, 9, and 10 and designated as *qPC_C-3*, *qPC_C-6*, *qPC_C-9*, and *qPC_C-10*. The *qPC_C-3* could be detected in both environments, while the other three QTLs could only be detected in 2016GZ.

### Candidate Gene Analysis of *qPC-1*

**FIGURE 3:**
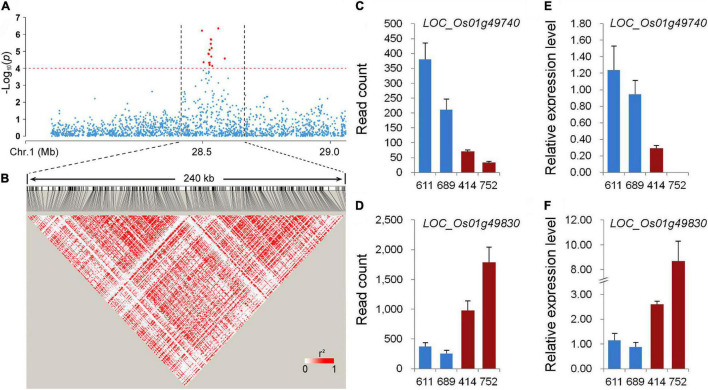
Candidate gene analysis of *qPC-1*. **(A)** Local Manhattan plot of GWAS for *qPC-1.* **(B)** Linkage disequilibrium (LD) heatmap around the peak. **(C,E)** The expression of *LOC_Os01g49740* in lines with white pericarp (accessions 611 and 689) and red pericarp (accessions 414 and 752) measured using RNA-seq and qRT-PCR, respectively. **(D,F)** The expression of *LOC_Os01g49830* in lines with white and red pericarp measured using RNA-seq and qRT-PCR, respectively.

Among the stably expressed and newly identified QTLs using the whole population in this study, *qPC-1* explained the largest phenotypic variation (Figures 2A,B and Table 1). The LD decay analysis in the QTL region indicated that an approximately 240 kb region (from 28.44 to 28.68 Mb on chromosome 1) was the putative region for *qPC-1* (Figures 3A,B). Based on release 7 of the MSU Rice Genome Annotation Project^2^ (Kawahara et al., 2013), there are 31 annotated genes within the *qPC-1* region (Supplementary Table 2). To reduce the number of candidate genes, we selected four lines with consistent flowering time, including two lines with white pericarp (accessions 611 and 689) and two lines with red pericarp (accessions 414 and 752) based on haplotype analysis of *qPC-1*, to conduct gene differential expression analysis. RNA-seq revealed that 17 genes were not expressed (Supplementary Table 2). Among the left 14 expressed genes, two genes, *LOC_Os01g49740* and *LOC_Os01g49830*, were differentially expressed between the two sets of contrasting lines (Figures 3C,D and Supplementary Table 2). Furthermore, qRT-PCR assays also confirmed these results (Figures 3E,F). However, the two genes exhibited the opposite expression patterns: the expression levels of *LOC_Os01g49740* in red pericarp lines were consistently and significantly lower than that in the white pericarp lines (*p* < 0.05), while *LOC_Os01g49830* in the red pericarp lines were significantly higher than that in white pericarp lines (*p* < 0.05).

### Analysis of the Allelic Variation of *Rc* Gene

**FIGURE 4:**
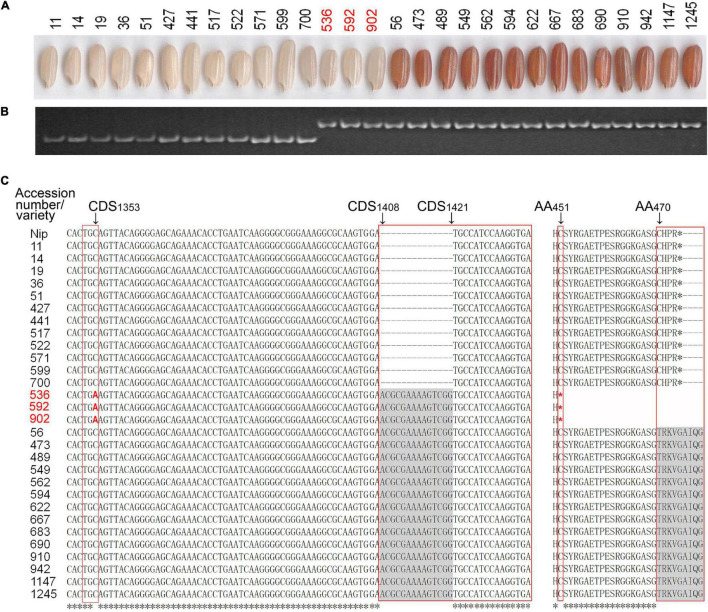
Analysis of allelic variation in *Rc* gene. **(A)** Exhibition of PC in the rice accessions. **(B)** Three rice accessions (accessions 536, 592, and 902) with white pericarp exhibited the same polymorphism as those with red pericarp in polyacrylamide gel electrophoresis. **(C)** Variation in the CDS region of the three and other rice accessions. The three rice accessions (accessions 536, 592, and 902) are highlighted in red fonts and the various regions are framed in red. Nip, *Nippobare*; CDS, coding sequence; AA, amino acid.

A previous study demonstrated that the white pericarp is mainly caused by a 14-bp deletion in exon 6 of the *Rc* gene compared with the brown or red pericarp (Sweeney et al., 2006). To investigate allelic variations of the *Rc* gene, we used a pair of Indel primers designed based on the 14-bp deletion (HM1, Supplementary Table 3) (Yang et al., 2017) to genotype the 100 lines with white pericarp and the 126 lines with colored pericarp. The PCR assay showed that the three lines (accessions 536, 592, and 902 from *Aus* sub-group) with white pericarp exhibited the same genotype as those with red pericarp (Figures 4A,B), suggesting that the *Rc* gene in the three lines with white pericarp has no 14-bp deletion. We further sequenced the *Rc* gene using four pairs of primers (Os07g0211500-1∼4, Supplementary Table 3) (Yang et al., 2017) in the three lines and other 26 lines, including 12 lines with white pericarp and 14 lines with red pericarp. In fact, the sequencing results revealed that the 12 lines with white pericarp had a 14-bp deletion (CDS_1408_–CDS_1421_) in the *Rc* gene compared with 14 lines with red pericarp, while the three lines did not have the 14-bp deletion in the *Rc* gene (Figure 4C) but had a C-to-A transversion at CDS_1353_ in their *Rc* gene compared with the 26 lines, which results in a termination codon (TGA) and a premature protein (Figure 4C). This result is consistent with the previous studies (Sweeney et al., 2007).

## Discussion

The PC in rice is a complex trait. Although it is well-known that proanthocyanidins and anthocyanins are responsible for PC and their biosynthetic pathway has been well-characterized, the genetic basis of PC in rice has not been well understood. In this study, we evaluated the PC of 442 rice accessions in two environments (2016GZ and 2018YJ) using different two phenotyping methods and GWAS of PC was conducted. Guangzhou belongs to the subtropical monsoon climate, while Yangjiang belongs to the subtropical maritime monsoon climate. The difference in light and temperature affects rice growth as manifested by the significant difference in growth periods of the rice accessions planted in Guangzhou (2016GZ) and Yangjiang (2018YJ) (*p* < 0.01, unpublished data). It is reported that light is an important factor affecting the expression of the flavonoid biosynthetic genes (Chen et al., 2013), suggesting that the difference in light and temperature may also lead to the variation in DPC of rice planted in the two places. Our results indicated that the white or colored pericarp of 442 rice accessions remain unchanged, while the DPC of the colored rice accessions varied across environments, suggesting that the presence and absence of PC in rice is a qualitative trait not affected by the environment, while the DPC of colored rice is a quantitative trait affected by the environment. In the whole population consisting of white and colored rice accessions, nine QTLs were identified using the phenotypes represented by presence and absence of pigmentation in pericarp, all of them were identical in both environments and determined the presence or absence of PC; while 10 QTLs were identified using DPC phenotypes. Interestingly, 8 out of 10 QTLs identified using DPC were the same as those identified using phenotypes of presence and absence of pigmentation, including *qPC-7*, which overlapped with the major gene *Rc*. After removing the white rice accessions, four QTLs for DPC were identified in the colored population. To our surprise, these four QTLs are totally different from the QTLs identified in the whole population using the phenotype of either DPC or the presence and absence of pigmentation. These results together suggest that there exist strong masking effects of major genes on QTLs with minor effects. This can explain why the QTLs for DPC identified in the colored rice population could not be detected in the whole population. Based on the coincidence of most of the QTLs identified in the whole population using either DPC phenotypes or the phenotypes represented by presence and absence of pigmentation and they were less affected by the environment, we believe that majority of QTLs identified in the whole population using DPC phenotypes could be still the QTLs determining the presence or absence of PC because of the masking effects of major genes. A previous study identified the same QTLs for DPC using a backcross-recombinant inbred line population consisting of white and colored rice lines in two different environments (China and Japan) (Dong et al., 2008), which might also be the evidence to support our inference. Therefore, it is critical to eliminate the masking effects of major genes before the QTLs controlling DPC in rice can be identified.

In total, 15 QTLs associated with PC in rice were identified in this study. Compared with the previous studies, 10 out of the 15 QTLs are first reported in this study, and the other five QTLs are co-localized with the previously identified QTLs or genes (Table 1), suggesting the reliability of the results and the diversity of the rice germplasm used in this study. The *qPC-7* overlapped with the *Rc* gene, which encodes a bHLH transcription factor regulating proanthocyanidin synthesis (Sweeney et al., 2006). The *qPC-8c* was co-localized with the previously reported QTL, whose candidate gene was *Os08g0301500*, which encodes a sucrose-phosphate synthase (Huang et al., 2010, 2011). The *qPC-8d* was co-localized with the previously reported QTL associated with grain color and flavonoid content (Shao et al., 2011). The *qPC_C-6* was co-localized with *OsC1*, an MYB transcription factor, which regulates anthocyanin biosynthesis in rice apiculus and sheath (Gao et al., 2011; Hu et al., 2020). Furthermore, *qPC_C-9* overlaps with *qDRC-9* for the degree of red coloration in rice pericarp (Dong et al., 2008).

Among the newly identified QTLs for PC using the whole population in this study, *qPC-1* could be detected across environments using different phenotyping methods and explain the largest phenotypic variation (Table 1). To identify the gene underlying *qPC-1*, we delimited it to a 240-kb region containing 31 putative genes based on the LD decay analysis (Figures 3A,B and Supplementary Table 2). Only two genes, *LOC_Os01g49740* and *LOC_Os01g49830*, were differentially expressed between the two sets of contrasting lines with white and red pericarps (Figures 3C–F) based on RNA-seq and qRT-PCR assays. *LOC_Os01g49740* encodes a domain of the unknown function (DUF) domain-containing protein. It has been reported that DUF proteins are involved in plant growth, development, and abiotic stress (Li et al., 2012; Ganie et al., 2017). *LOC_Os01g49830* encodes a Related to ABI3/VP1 (RAV) transcription factor, which belongs to the subfamily member of the AP2/ERF family. Himi et al. (2011) isolated three F3H genes, which were highly expressed in red grains and red coleoptiles but not in white tissues in wheat and found an RAV1 binding site in their promoters. Recently, two groups simultaneously reported that RAV transcription factors were involved in the regulation of flavonoid biosynthesis in fruit plants (Zhang et al., 2020; Zhao et al., 2020). Zhao et al. (2020) identified three AP2/ERF transcription factors (CitRAV1, CitERF32, and CitERF33), which positively regulate the expression of *CHI* (*CitCHIL1*) in citrus. CitERF32 and CitERF33 activated the transcription by directly binding to the promoter, while CitRAV1 formed a transcription complex with CitERF33 to strongly enhance the activation efficiency and flavonoid accumulation. The other study reported that *FaRAV1* stimulated anthocyanin accumulation in strawberries by directly activating transcription of *FaMYB10* and the structural genes of anthocyanins biosynthesis, *CHS*, *F3H*, *DFR*, and *GT* (Zhang et al., 2020). The *CHS*, *CHI*, *F3H*, and *DFR* are the important structural genes in pathways of flavonoid biosynthesis (Winkel-Shirley, 2001; Kim et al., 2008). Based on gene differential expression analysis in this study and the gene functions in the previous studies, *LOC_Os01g49830* is more likely the candidate gene underlying *qPC-1*. However, further transgenic experiments are needed to confirm its function on PC in rice.

Among the four QTLs for DPC identified in the colored population, *qPC_C-3* explained the largest phenotypic variation and was the only QTL detected across environments. Therefore, it has a great potential value in rice breeding for DPC. By analyzing the distribution of nucleotides at the most significant SNP position of *qPC_C-3* in the rice accessions with different DPC, it was found that most of the rice accessions (78.6%) with dark PC (DPC 5-7) harbor the “T,” while only 13.8% of the rice accessions with light PC (DPC 2-4) harbor the “T.” The result provides valuable information for the development of the SNP marker for *qPC_C-3* selection in rice breeding.

*Rc* is the most important regulatory gene involved in the proanthocyanidin synthesis in rice pericarp. Compared with red pericarp, the most white pericarp is caused by a loss-of-function allele, *rc*, characterized by a 14-bp deletion in exon 6 of *Rc* gene (Furukawa et al., 2006; Sweeney et al., 2007). In this study, an allele of the *Rc* gene in white pericarp without a 14-bp deletion in exon 6 was identified in the three Aus accessions. Further sequence analysis showed that a C-to-A transversion at CDS_1353_ exists in the allele of the *Rc* gene, which causes early termination of translation (Figure 4). Previous studies also identified this allelic variation in *Rc*, namely *Rc-s*, which is mainly found in the Aus subpopulation and produces white pericarps but light-red pericarps in some genetic backgrounds (Sweeney et al., 2006, 2007). Recently, Wang et al. (2016) identified an Aus-specific QTL, *qPc10*, and indicated that the presence or absence of *qPc10* resulted in light-red or white pericarps, suggesting that interaction between *Rc* alleles and other loci may also affect the PC in different lineages. Except for *rc* and *Rc-s*, the *rc-gl* characterized by an A-to-T substitution at CDS_1207_ in *Rc* also results in a premature stop-codon and a white pericarp in the domesticated African rice (Gross et al., 2010). Furthermore, *Rc^r^* and *Rc-g*, having a G base deletion at the 20 or 44 bp upstream of the 14-bp deletion, cause reverse mutation to red pericarp (Brooks et al., 2008; Lee et al., 2009). This inspires us that these mutant regions are potentially important as *cis*-regulatory regions. It remains to be studied whether mutations in other genes also cause a difference in PC.

## Data Availability Statement

The original contributions presented in the study are included in the article/Supplementary Material, further inquiries can be directed to the corresponding authors.

## Author Contributions

WY, BL, and SZ conceived and designed the research. WY, LC, and JZ conducted the experiments, performed data analysis, and wrote the manuscript. JW, WL, TY, JD, YM, LZ, JC, and WW participated in material development, sample preparation, and data analysis. WY and BL drafted proposals and corrected the manuscript. All authors read and approved the final manuscript.

## Conflict of Interest

The authors declare that the research was conducted in the absence of any commercial or financial relationships that could be construed as a potential conflict of interest.

## Publisher’s Note

All claims expressed in this article are solely those of the authors and do not necessarily represent those of their affiliated organizations, or those of the publisher, the editors and the reviewers. Any product that may be evaluated in this article, or claim that may be made by its manufacturer, is not guaranteed or endorsed by the publisher.

## Footnotes

1
https://www.ncbi.nlm.nih.gov/tools/primer-blast/

2
http://rice.plantbiology.msu.edu/

## Supplementary Material

Supplementary Figure 1 The linkage disequilibrium (LD) decay in the 442 rice accessions.
