# Supplementary figures and images for: Genome-Wide Association Study of Pericarp Color in Rice Using Different Germplasm and Phenotyping Methods Reveals Different Genetic Architectures
Source: Front Plant Sci. 2022 Mar 9;13:841191. doi: 10.3389/fpls.2022.841191 (PMC8959774; doi:10.3389/fpls.2022.841191)

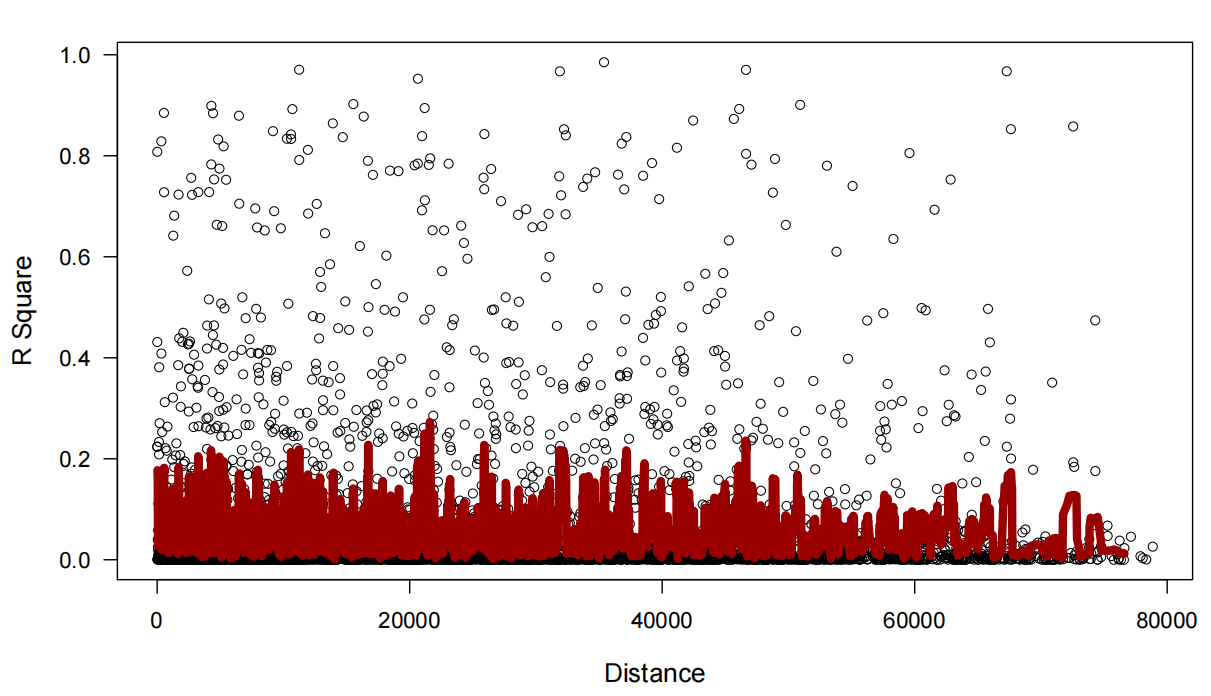

Supplement: Supplementary Figure 1 — The linkage disequilibrium (LD) decay in the 442 rice accessions. [file Image_1.JPEG]
